# Traversing behavior of tumor cells in three-dimensional platforms with different topography

**DOI:** 10.1371/journal.pone.0234482

**Published:** 2020-06-10

**Authors:** Z. Y. Liu, W. G. Zhang, S. W. Pang

**Affiliations:** 1 Department of Electrical Engineering, City University of Hong Kong, Hong Kong, China; 2 Center for Biosystems, Neuroscience, and Nanotechnology, City University of Hong Kong, Hong Kong, China; Politecnico di Milano, ITALY

## Abstract

Three-dimensional polydimethylsiloxane platforms were developed to mimic the extracellular matrix with blood vessels by having scaffolds with micropatterns, porous membrane and trenches. Precisely controlled physical dimensions, layouts, and topography as well as different surface chemical treatments were applied to study their influences on nasopharyngeal carcinoma cell (10–15 μm in diameter) migration in mimicked platforms over 15-hour of time-lapse imaging. By placing the pores at different distance from the edges of the trenches, pores with different trench sidewall exposures and effective sizes were generated. Pores right next to the trench sidewalls showed the highest cell traversing probability, most likely related to the larger surface contact area with cells along the sidewalls. Straight grating oriented perpendicular to trenches below the top layer increased cell traversing probability. Pore shape as well as pore size influenced the cell traversing probability and cells could not traverse through pores that were 6 μm or less in diameter, which is much smaller than the cell size. Trench depth of 15 μm could induce more cells to traverse through the porous membrane, while shallower trenches impeded cell traversing and longer time was needed for cells to traverse because 3 and 6 μm deep trenches were much smaller than cell size which required large cell deformation. Hydrophobic surface coating on the top layer and fibronectin in pores and trenches increased the cell traversing probability and reduced the pore size that cells could traverse from 8 to 6 μm, which indicated that cells could have larger deformation with certain surface coatings.

## Introduction

Cancer has caused many deaths for all ages around the world. Nasopharyngeal carcinoma (NPC), compared with other cancer types, is unique in its population distribution, pathology, and diagnose [1–4]. It shows a remarkable geographical distribution in southern China and south-east Asia and occurs in younger patients [4–7]. Among the different cancer cell migration behaviors, circulating tumor cells were the most harmful as they could cause cancer invasion, secondary tumors sites and finally lead to patient death [8]. Migration of cells in circulating system has been considered as the key in understanding cancer metastasis and circulating tumor cells [9–12]. Migration behaviors of cancer cells in two-dimensional system *in vitro* have been studied including cell migration on micropatterns, under confinement, and cell separation [13–17]. However, there is still limited understanding of cancer cell invasion because the microenvironments used for the studies were quite different from the highly complicated extracellular matrix (ECM) *in vivo*.

Thus, studying tumor cell behaviors in three-dimensional (3D) ECM that mimicks microenvironment *in vivo* is very important to solve the many unanswered questions in cancer cell invasion and metastasis. Cell behaviors in *in-vitro* ECM made from gel or collagen had been studied including cell-matrix adhesions, cell motility, cell invasion, cell migration mode, and mechanotransductive signaling [18–23]. However, the effects of the matrix environment on cell migration or cancer invasion are not clear because the limited control of gel or collagen formation [20, 24], resulting in poorly defined pore size or other biophysical parameters in these 3D gel or collagen matrix systems [20, 23]. Therefore, a better controlled ECM with precisely defined microenvironment will be needed to understand the mechanisms of the cell intravasion and extravasion through the blood vessels. Previous studies have shown cell migration and invasion dynamics in microfluidic platforms with complex microchannels [25–27]. The results revealed that the dimensions and layouts of the microchannels are critical in influencing cell transgression dynamics and invasion probabilities.

Herein, we proposed a polydimethylsiloxane (PDMS) 3D matrix to mimic the ECM topography around blood vessels, the porous epithelial membrane, and the underlying blood vessels as an *in-vivo* microenvironment to study tumor metastasis in this work. With this 3D matrix, topography properties such as pore size and pore shape could be precisely controlled. Besides, this transparent 3D biomimetic platform could allow real-time observation of the entire cell migration process including migration behavior and mode before and after cell traversing through porous membrane, which is similar to the invasion process of cancer cell *in vivo*. In this paper, we will focus on the factors influencing the NPC43 cancer cell traversing behavior through porous membrane. Physical factors including topography on top layer, pore size of membrane in middle layer, and trench depth in bottom layer, and fibronectin (FN) coating on different layers will be investigated. The results could lead to the control of tumor cell migration and traversing through membrane.

## Experimental

### Technology for 3D biomimetic platforms

**Fig 1:**
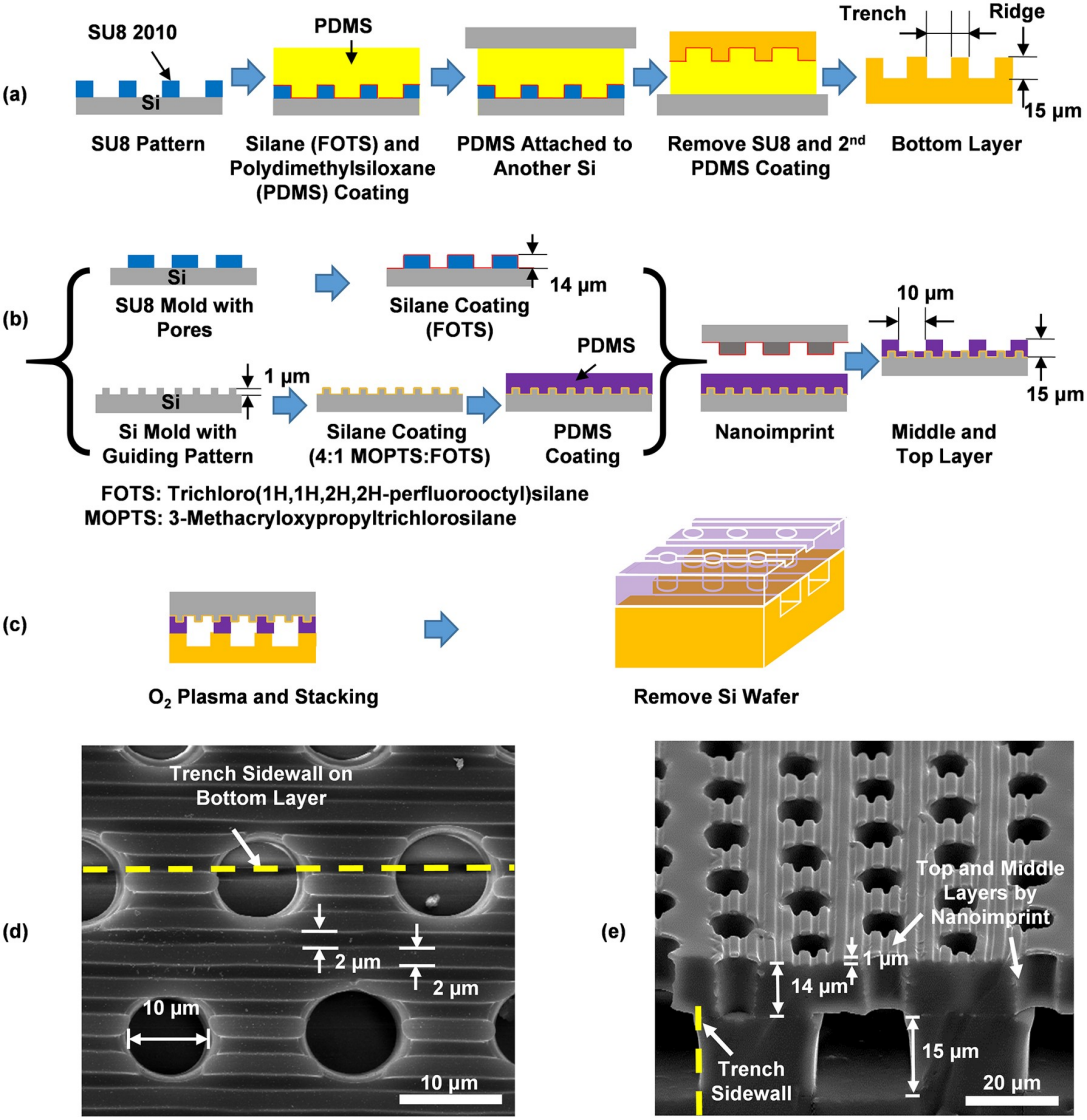
Fabrication of 3D biomimetic platform. (a) Bottom layer by demolding PDMS from SU8 mold. (b) Middle and top layers fabricated by reversal nanoimprint using SU8 and Si molds with guiding grating. (c) Stacking two layers after O_2_ plasma treatment. Micrographs of 3D platform with (d) top and (e) cross sectional views. Grating was parallel to trench orientation. Platform had 2/2 μm trench/ridge and 1 μm deep gratings on top, 10 μm dia. and 14 μm deep pores in middle, 30 μm wide and 15 μm deep trenches in bottom.

In the work, three-layer platforms with shallow topographical structures on the top layer, porous membranes in the middle layer, and trenches in the bottom layer were designed as shown in Fig 1. The fabrication technology involved multiple imprints. All the layers were made from biocompatible material, PDMS. The bottom layer with the trenches to mimic blood vessels was replicated from a Si mold with trenches etched by dry reactive ion etching as shown in Fig 1(a). The middle and top layers of the platforms were imprinted simultaneously from a pore-patterned SU-8 mold coated with trichloro(1H, 1H, 2H, 2H-perfluorooctyl) silane (FOTS) and a grating-pattern Si mold coated with a silane mixture of FOTS and 3-methacryloxypropyltrichlorosilane as shown in Fig 1(b). The different silane coatings provided different surface energy, resulting in the replicated middle and top PDMS layers to be formed as a porous membrane with grating topography. Then, the middle and bottom layers were both treated with an O_2_ plasma and these combined layers were bonded to a bottom trench layer right after the plasma treatment and baked for 10 min at 80°C. The bonded platform was then peeled off from the Si mold as shown in Fig 1(c) because the bonding strength of the three-layer PDMS platform was higher than the adhesion strength between the PDMS platform and the Si mold as designed by the silane coatings. Fig 1(d) and 1(e) showed the scanning electron micrographs of the fabricated platform with the top layer grating parallel to the bottom trench orientation. This 3D platform consisted of 2/2 μm trench/ridge and 1 μm deep grating on top, 10 μm diameter (dia.) and 14 μm deep pores in the middle, and 30 μm wide and 15 μm deep trenches in the bottom.

### Physical and chemical designs

In order to control the tumor cell traversing behaviors by the physical patterns on the 3D platforms, topographical patterns with different dimensions and orientations on different layers were designed. Different offsets between the edge of pores in the middle layer and the sidewalls of trenches in the bottom layer were investigated. Grating orientation and grating bending angles on the top layer were used to guide cell migration towards the pores. Circular, triangular, and square pores with the same area in the middle layer were introduced to study the pore shape preference of cells when they traversed through porous membranes.

Effects of pore size (from 3 to 20 μm) on cell traversing probability was used to study cell deformation capability. Circular and elliptical pores with the same dia. of 10 μm (long axis length of 25 μm for elliptical pores) were also designed to investigate cell migration mode and traversing location preference. To understand the effects of blood vessel size on tumor cell traversing through porous membrane, trench depths in bottom layers were changed from 3, 6, 15, and 30 μm. In addition, FN (50 μg/ml in DI water, Sigma-Aldrich, MO, USA) was coated in the middle and bottom layers, and 0.2% Pluronic F-127 (Sigma-Aldrich, WI, USA) was coated on the top layer to study the chemical coating effects on cell traversing through pores.

### Cell culture and assays

Stable cell line of nasopharyngeal carcinoma (NPC43) cells infected with Epstein-Barr virus (EBV) were used for this study [28]. The EBV-positive NPC43 cell line was obtained using patient NPC tissues with Rho-associated coiled-coil containing kinases (ROCK) inhibitor (Y-27632) added in culture medium [28]. The culture medium was Roswell Park Memorial Institute 1640 medium (Gibco), supplemented with 10% fetal bovine serum (FBS, Gibco) and 1% antibiotic antimycotic (Gibco; 100 units/ml penicillin G sodium, 100 mg/ml of streptomycin, and 0.25 mg/ml of amphotericin B). The cells were incubated at 37 °C in a 5% CO_2_ incubator. The medium was changed every 2 days and 0.2% 2 mM ROCK inhibitor Y-27632 (25 mg, ENZO) was added at the same time.

Some cells did not move or moved much more slowly, or even became dead. Some cells divided during the imaging period. The doubling time for NPC cells was 24 h and most of the cells would divide by then. After cell division, the cells often moved faster and in opposite directions. In this study, cells were seeded on the platform and placed in the incubator for 5 h and observed under time-lapse imaging for additional 15 h. If cells died or divided during this period, they were not included in the analysis.

### Time-lapse imaging

The three-layer platforms with different physical dimensions were bonded on 35 mm glass bottom confocal dishes (SPL Life Sciences) using an O_2_ plasma treatment for 1 min with a gas flow rate of 20 sccm, a chamber pressure of 80 mTorr, and RF power of 60 W. The O_2_ plasma was used again to prepare the platform surface for better cell seeding. Typically, NPC43 cells were seeded at a density of 1 × 10^5^ cells/cm^2^ on the top layer of the three-layer platforms. The NPC43 cells were incubated for 5 h after seeding at 37°C and in 5% CO_2_ in a humidified incubator. After initial attachment, the mediums for NPC43 cells were replaced by a 1:1 mixture of NPC43 culturing medium and CO_2_-independent medium (Invitrogen 18045–088) supplemented with 10% FBS, antibiotic-antimycotic (100 U/ml of penicillin, 100 mg/ml of streptomycin, and 0.25 mg/ml of amphotericin B) and 2 mM alanyl-L-glutamine. The cells were imaged using a scanning Nikon Eclipse Ni upright microscope equipped with an incubation chamber at 37 °C. Image capturing was carried out every 5 min over a period of 15 h. All the data were from at least 2 independent assays. The exact numbers of assay are indicated in each figure caption.

Different chemical treatments (FN and Pluronic) were applied on the 3D platforms. To coat FN in the middle and bottom layers and Pluronic on the top layer, FN was flowed into the bottom trench layer through the inlet on the porous membrane for 12 h, followed by immersing the platform in Pluronic for 30 min to inhibit cell attachment on the top surface. To have a hydrophobic surface on the top layer, only FN was flowed into the bottom trench layer through the inlet on the porous membrane for 12 h. To provide FN coating in middle and bottom layers but with a hydrophilic surface on the top layer, the 3D platforms bonded on glass dishes were treated with an O_2_ plasma again and FN was flowed into the bottom trench layer through the inlet. After the surface treatment, 70% ethanol, 0.1% phosphate-buffered saline (PBS), and culture medium for NPC43 cells were separately used to wash the platforms, and NPC43 cells were seeded on the platforms right after medium washing.

### Data analysis

NIH ImageJ (version 1.48) software package with manual tracking plugin and image stabilizer plugin was used to track cells that did not divide or physically interact with other cells during the 15 h imaging period. Cell trajectories and protrusion length were analyzed by Microsoft excel and Origin 8.0. Offset between pore edge and trench sidewall meant the distance between the closest edge of the pores and the nearest sidewall of the trenches in the bottom layer. Positive offset indicated the pore was away from the trench below on the suspended membrane and negative offset meant the pore had overlapped with the trench sidewall below.

Protrusion length was defined as the distance between the edge of the cell and the protrusion tip. Traversing time was defined as the time interval between cell seeding and cell starting to traverse through the porous membrane. Traversing probability was calculated by dividing the cell number traversing to the bottom trench layer by the number of total cells around the pores when confocal imaging began. Cell morphology was observed by optical imaging and scanning electron microscopy (SEM). Statistical difference between groups was tested using one-way analysis of variance (ANOVA) with Dunnett’s post-hoc analysis. All the results are presented as mean ± standard error of the mean. All quantitative data presented in this paper were based on at least three runs and a large number of cells, mostly more than 30 cells and up to 346 cells. The only exceptions were for the cases with pore size smaller than 6 μm and the square shape pores, which had two runs because the traverse probability was zero and no cells could get through the pores. Even for those with two runs, the cell numbers were at least 25 and up to 57.

### Scanning electron microscopy

After NPC43 cells were cultured on PDMS platforms for 20 h as described above, they were rinsed with the 1% PBS at 37 °C, followed by fixation with 4% paraformaldehyde for 30 min. Fixed cells were dehydrated through a series of increasing ethanol concentration (30%, 50%, 70%, 80, 90%, 95%, and 100%). The dehydration was completed by critical point drying and the transitional gas was carbon dioxide. After coated with a thin Au film by evaporation, the samples were imaged under an environmental scanning electron microscope (Hitachi, SU5000).

## Results

### Cell traversing probability dependence on pore size

**Fig 2:**
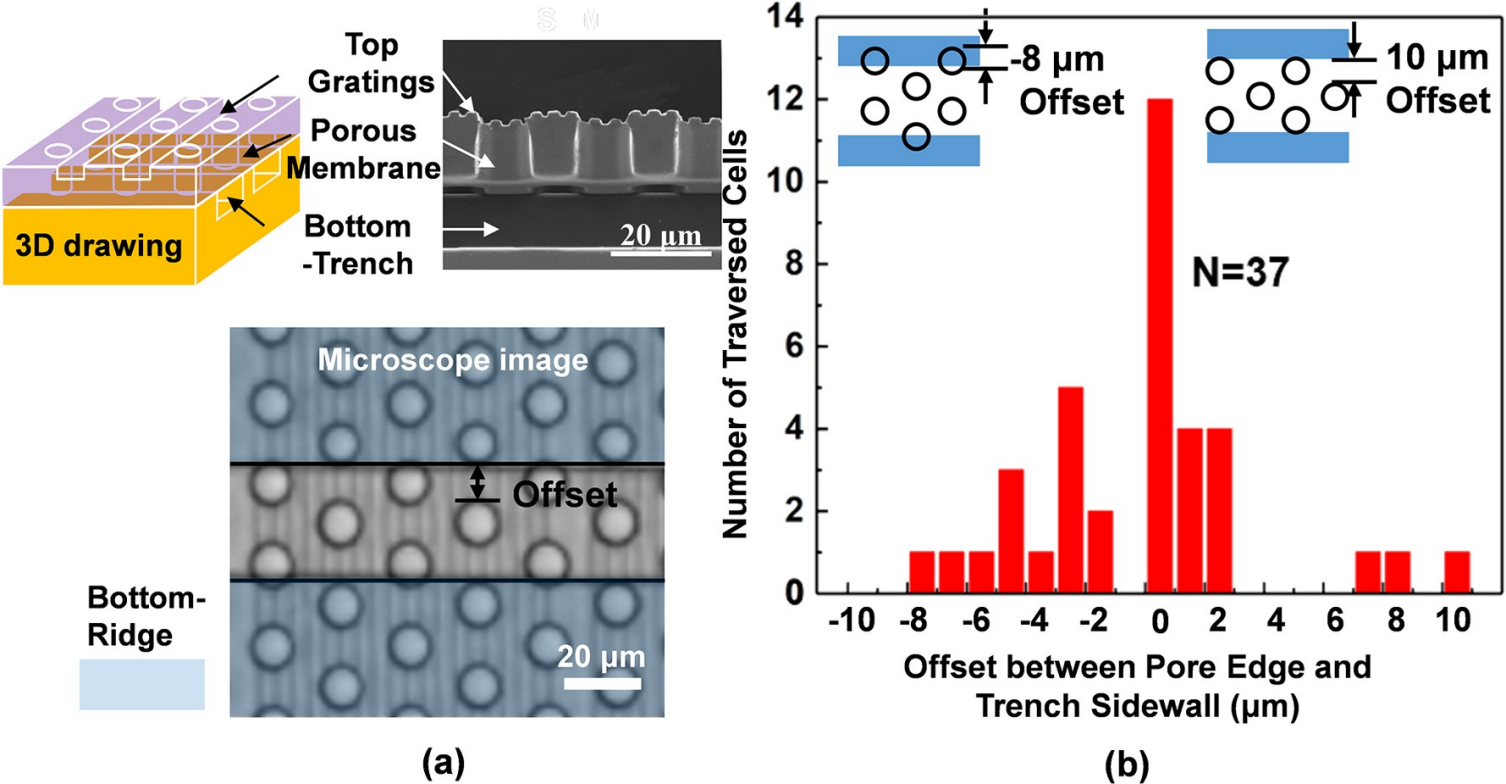
3D biomimetic platform and NPC43 cell traversing probability. (a) 3D biomimetic platform with 2/2 μm trench/ridge and 1 μm deep gratings on top, 10 μm dia. and 14 μm deep pores in middle, and 30 μm wide and 15 μm deep trenches in bottom. (b) Number of NPC43 cells traversed though pores with different offset between pore edge and trench sidewall (N = 37 in 6 runs).

Fig 2 shows the number of NPC43 cell traversing through the pores to the bottom trench layer as a function of the offset between the pore edge and the trench sidewall. Fig 2(a) presents three images including a 3D drawing, a cross-section-view of the SEM corresponding to the 3D drawing, and a top-view of the platform with the top grating perpendicular to the bottom trench. The total number of cells traversing to the bottom layer was 37 in 6 runs. When the offset was zero (pore edge right next to the trench sidewall), the probability for cells to traverse through the porous membrane to the bottom trench layer was highest. For both positive and negative offsets, the traversing probability decreased as the offset became larger, as shown in Fig 2(b). Cells could traverse through the pores right next to the trench sidewalls more easily because the sidewalls in bottom layer provided more contact surface for the cell lamellipodia to spread out and migrate. Being able to contact the trench sidewalls would promote cell migration through the pores to reach the bottom surface of the trenches. The lamellipodia would extend straight through the porous membrane onto the trench sidewalls without any bending or turning. On the other hand, it would be much more difficult for the lamellipodia to bend 90° in order to reach the bottom layer of the porous membrane. Even after the bending, the lamellipodia would be away from the trench sidewalls or the trench bottom surface, which resulted in much more limited surface contact for cell migration. Thus, with a larger positive offset, NPC43 cells had to migrate a longer distance in order to contact the sidewall of the bottom trench after they traversed through the pores, resulting in a lower number of traversed cells. When the offset was negative, the effective pore size was smaller because part of the pore was blocked by the membrane on top. Cells had to squeeze through the smaller pore area in order to traverse to the bottom trenches. Near the edge of the pores, cells sensed the large local curvature formed between a flat surface and a vertical sidewall. This local curvature near the edge could attract the cells into the pores as suggested by previous study [29]. When the negative offset value was -10 μm, the pore area became zero and the entire pore was blocked by the membrane and no cells could traverse to the bottom trenches. Based on above results, most of the data analysis of cell traversing behaviors will be all carried out when the offset was zero, when the pore was right next to the trench sidewalls.

### Topography on top layer regulated tumor cell traversing probability

**Fig 3:**
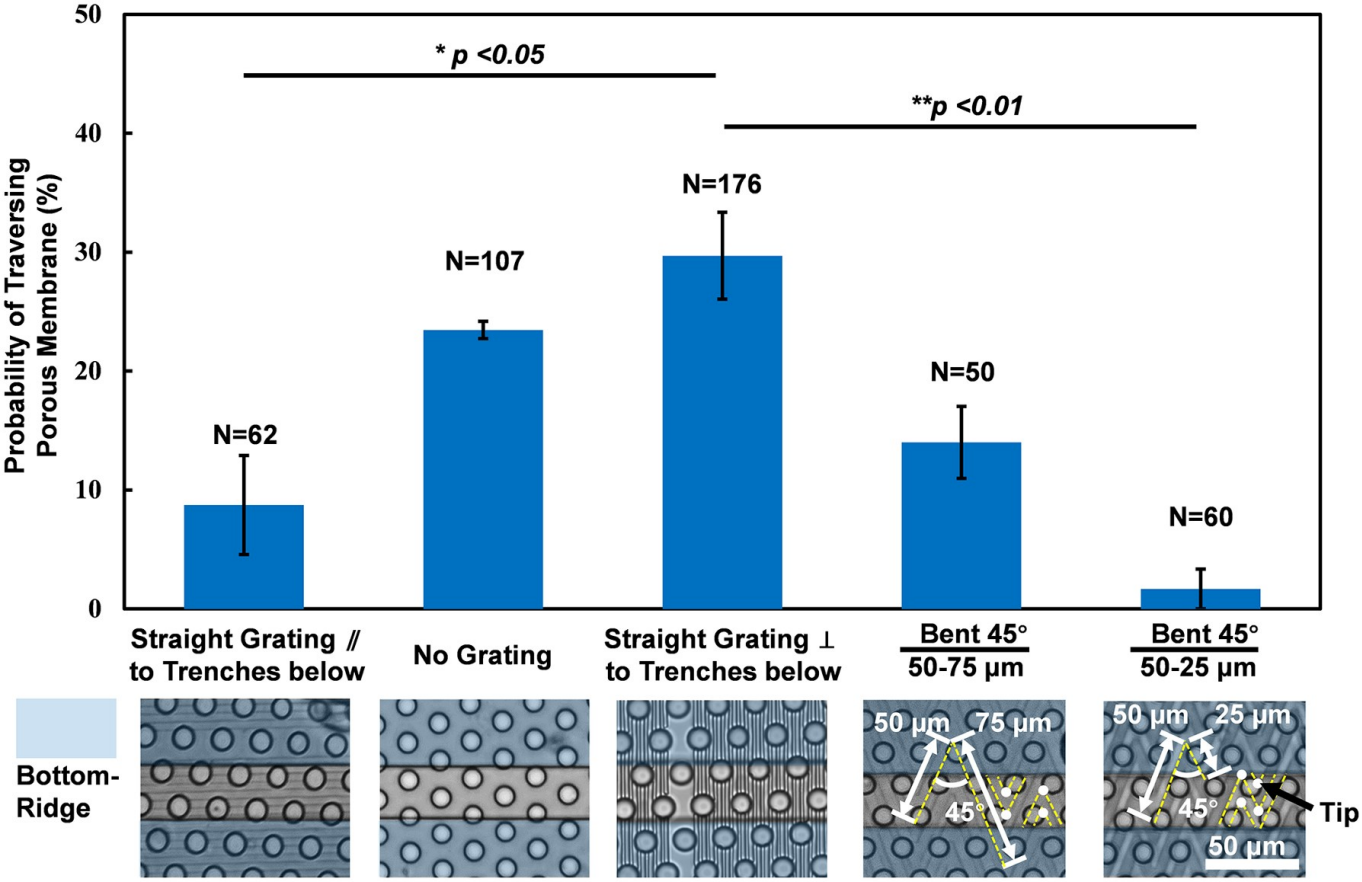
Cell traversing probability dependence on grating orientation and bending angle. Dependence of NPC cell traversing probability on grating orientation and grating bending angle. Three-layer platforms consisted of guiding gratings (straight and bent), porous membrane, and trenches below. (2/2 μm trench/ridge and 1 μm deep straight/bent gratings on top, 10 μm dia. and 14 μm deep pores in middle, and 30 μm wide and 15 μm deep trenches in bottom. 6 runs for gratings perpendicular to trench below and 3 runs for other conditions. *p <0.05 when comparing grating orientation and **p <0.01 when comparing grating bending angle.

In order to guide more cells through the pores and into the trenches below, grating orientation relative to the trench orientation and grating bending angle were investigated. Fig 3 shows the dependence of NPC cell traversing probability on grating orientation and grating bending angle. 6 runs each were carried out for cell migration on platforms with top gratings perpendicular to the bottom trenches and 3 runs each for other conditions. It had been reported that gratings could guide cell migration [30–32]. Thus, when the grating orientation was perpendicular to the trench orientation as shown in middle bar of Fig 3, cells would be guided through the pores towards the trench sidewalls. As discussed before, more cells could traverse through the pores when cells could contact the trench sidewalls. Consequently, more cells could traverse through the pores when the gratings were perpendicular to the trench orientation in the bottom layer. When no grating was on the top layer as shown in the second bar of Fig 3, cell migration direction was random and less cells could migrate through the pores towards the trench sidewalls below, which lowered the cell traversing probability. When grating orientation was parallel to the trench orientation in the bottom layer as shown in the first bar of Fig 3, more cells were guided along the grating orientation and not towards the trench sidewalls below, which further reduced the cell traversing probability.

As shown in Fig 3, less cells on platforms with grating angle of 45°, compared to straight grating, could traverse through the porous membrane to the bottom trench layer. It could be due to the bent gratings had less intersections with trench sidewalls compared to straight gratings that were perpendicular to trenches below. It had been shown that bent gratings had the effect of reversing cell migration direction [16, 30]. When segment length of the bent grating became shorter, more cells migrated in the reversed direction so less cells could move towards the trench sidewalls. Therefore, the cell traversing probability was the lowest when bent grating with shorter segment length was formed on the top layer. S1 Video shows a NPC43 cell on the platform with straight grating and it moved around the pore. However, the cell on the platform with bent grating moved around the bent tip and did not move around the pore. Less cells could move around the pores and traverse into the bottom trenches in the platforms with bent gratings on top due to the limited intersections with the trench sidewalls.

### Effects of pore shape and size on tumor cell traversing probability

**Fig 4:**
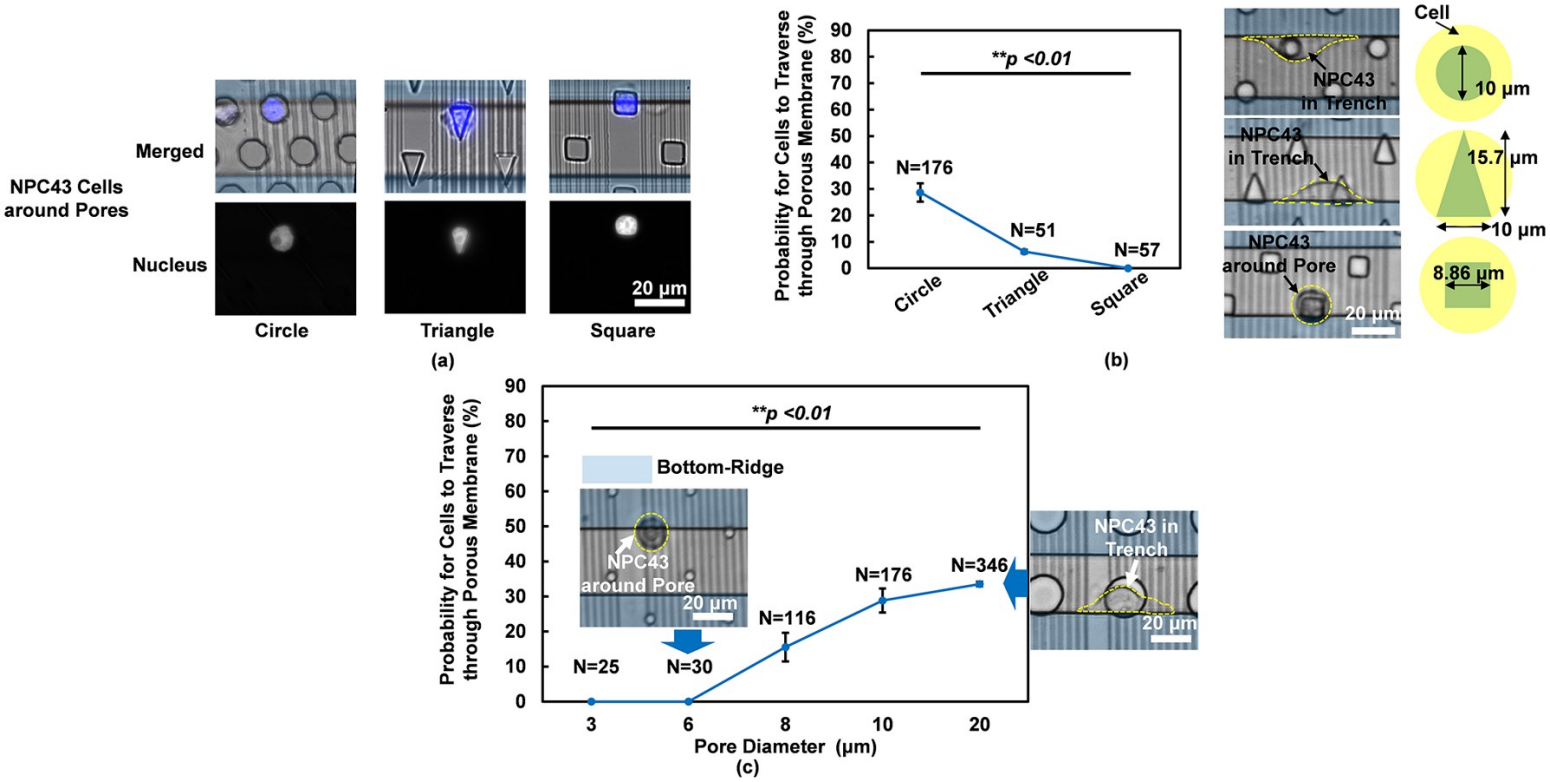
Cell traversing probability through pores with various size and shape. (a) Cell nucleus shape around circular, triangular, and square pores before cell traversing. (b) Pore shape changed cell traversing probability. (c) Cell traversing probability through porous membrane increased with size of circular pores. (2/2 μm trench/ridge and 1 μm deep gratings on top, 14 μm deep pores in middle, and 30 μm wide and 15 μm deep trenches in bottom. 6 runs for circular shape pores, 2 runs for square shape pores and 3/6 μm dia. circular pores, and 3 runs for other conditions. **p <0.01 when comparing pore shape and pore diameter.

There are endothelial cells that will form passage openings with different shapes around the blood vessels *in vivo*. In order to study the shape preference of cancer cells when traversing through a porous membrane, different pore shapes including circle, square, and triangle with the same area were designed as shown in Fig 4(a) and 4(b). 6 runs were carried out for circular shape pores and 3 runs for other conditions. Fig 4(a) shows that cell nuclei passing through pores with different shapes were deformed to the pore shape, and cells in the circular pores had the least deformation. Thus, cell traversing probability through circular pores was significantly higher than the other pore shapes as shown in Fig 4(b). For cells with the same area, square pores had the shortest edge compared to the circular or triangular pores. As a result, cell traversing probability was the lowest through the square pores.

Fig 4(c) shows that cell traversing probability decreased as the circular pore size diminished from 20 to 3 μm. 6 runs were carried out for 10 μm dia. pores, 2 runs were for 6/3 μm dia. pores, and 3 runs for other conditions. Smallest pore size that cells allowed NPC43 cells to traverse through was 8 μm. It had been reported that cell motility decreased as pore size became smaller in hydrogel matrix or collagen scaffolds [18, 19, 33]. Most of these studies indicated that smaller pore size limited cell membrane protrusions. Besides, cell migration mode could change from mesenchymal to amoeboid mode when cells traversed through pores. This is consistent with our study as shown in Fig 4(c), in which cells showed a round shape (amoeboid mode) around pores before traversing to the bottom trench layer and they changed to an elongated shape along the trench sidewall (mesenchymal mode) after fully traversed to the bottom layer. The smaller the pore size, the rounder the cells became. Thus, the amoeboid migration mode was adopted by cells as the pore size was small. However, when pore size was 20 μm, larger than the cell size, the cells had the ability to elongate and move in the mesenchymal migration mode when there was more space for cells to spread and extend their protrusions. Typically, cells migrate in amoeboid mode have higher motility than those moving in mesenchymal mode.

### Mesenchymal migration mode and guidance effects in elliptical pores

**Fig 5:**
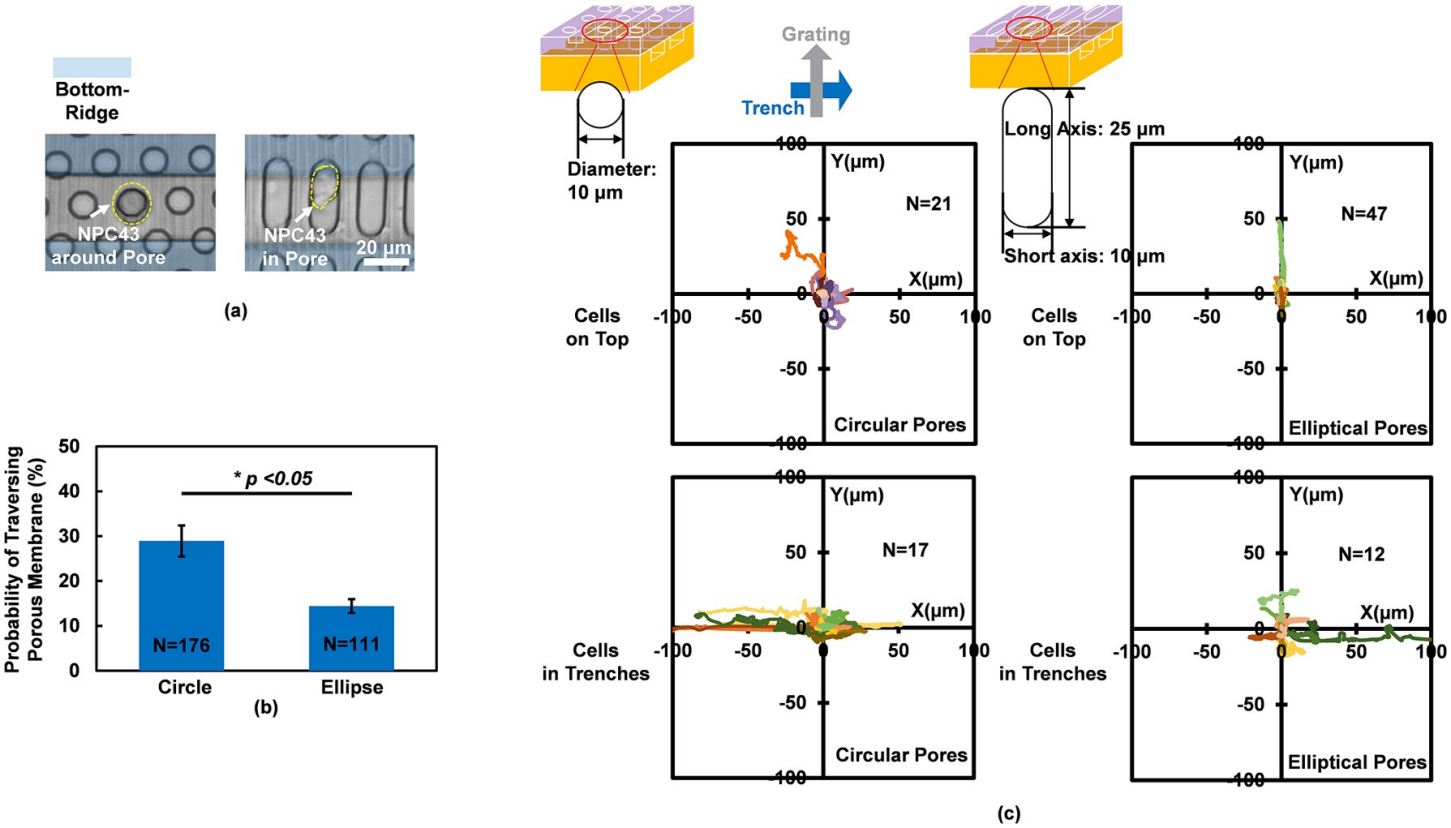
Cell traversing probability and migration trajectories on circular and elliptical pores. (a) Cells on top layer of 3D platforms with circular and elliptical pores. (b) Cell traversing probability through circular and elliptical pores. (c) Migration trajectories for cells on top and traversed through circular and elliptical pores. (2/2 μm trench/ridge and 1 μm deep gratings on top, 10 μm dia. and 14 μm deep pores in middle, and 30 μm wide and 15 μm deep trenches in bottom. 6 runs for circular shape pores and 3 runs for other conditions. *p <0.05 when comparing pore shape.

Elliptical pores with larger area were compared to circular pores to study cell migration behavior when there was more space for cell spreading. Fig 5(a) shows cell morphologies in the 10 μm dia. circular pore (6 runs) and elliptical pore (3 runs) with 10 and 25 μm short and long axes, respectively. Compared with rounded cell body in the circular pore, cell had more elongated shape and extended protrusion along the long axis sidewall of elliptical pore, which indicated that cell could move in the mesenchymal migration mode. It was observed that cell traversing probability was significantly lower in 3D platforms with elliptical pores compared to circular pores as shown in Fig 5(b). Cell trajectories on the top layer and in the bottom layer were shown in Fig 5(c). Cells on top of the elliptical pore membrane without traversing to the bottom trench layer migrated along the long axis sidewalls of the elliptical pores due to the migration guidance along the long axis. On the other hand, cells on top of the circular pore membrane without traversing to the bottom trench layer migrated randomly around the pores. However, cells migrated all along the trench sidewalls After the NPC43 cells traversed to the bottom trench layer, all cells migrated along the trench sidewalls regardless of whether the pores were circular or elliptical in the membranes. The results indicated that the larger size of the elliptical pores did not increase the traversing probability compared to the circular pore due to the modified cell shape along the long axis sidewalls which led to a different migration mode.

### Trench depth affected cell traversing probability and time interval before traversing

**Fig 6:**
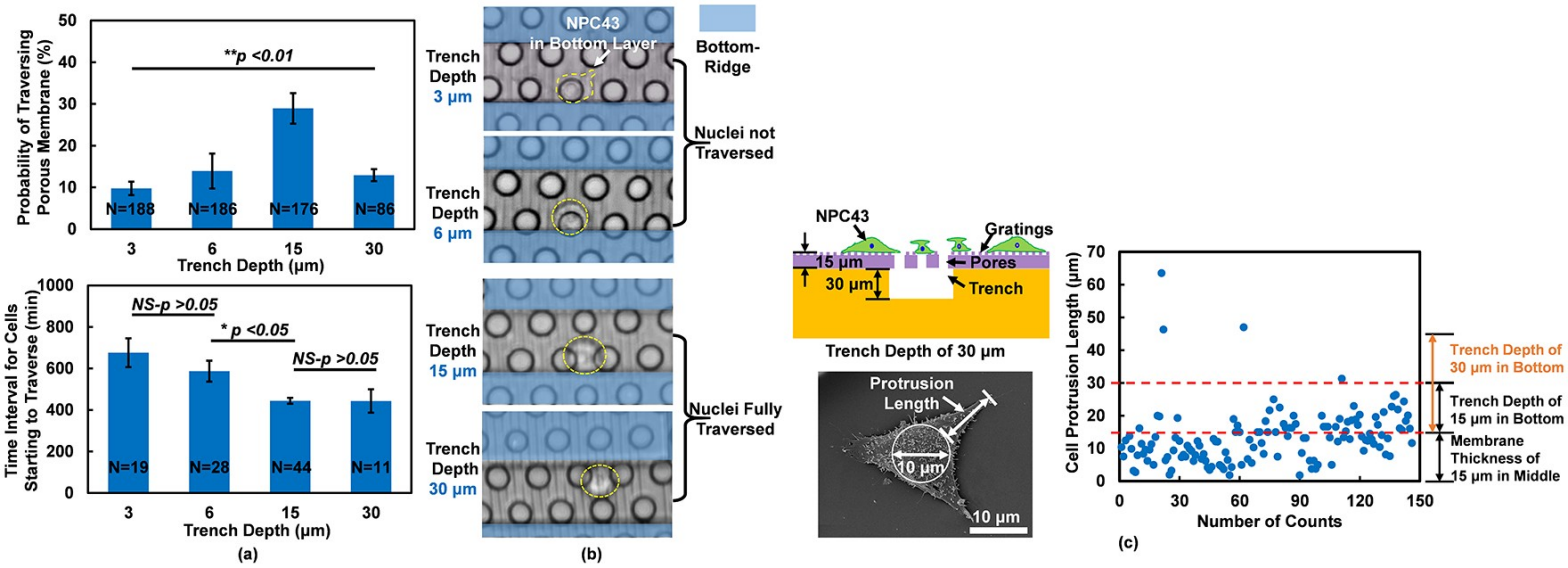
Effect of trench depth on cell traversing probability and time interval before traverse. Effects of trench depth on (a) cell traversing probability and time interval for cells starting to traverse, (b) cell morphology, and (c) cell protrusion length (N >50). (2/2 μm trench/ridge and 1 μm deep gratings on top, 10 μm dia. and 14 μm deep pores in middle, and 30 μm wide and 3, 6, 15, and 30 μm deep trenches in bottom. 6 runs for 15 μm deep trenches and 3 runs for other conditions. **p <0.01 for traversing probability dependence on trench depth and *p <0.05 for time interval dependence on trench depth.

Trench depth in the bottom layer of the 3D platform determined the available space for cells to migrate into, and it could affect the cell traversing probability. The cell traversing probability was the highest for 15 μm deep trenches, and it decreased for shallower or deeper trench depth as shown in Fig 6(a). Moreover, the time interval for cells to start to traverse through porous membrane decreased as the trench depth decreased from 3 or 6 μm to 15 or 30 μm. When the trench depth was 3 or 6 μm, it was smaller than the cell size and cells had difficulty in squeezing through the small pores. Thus, the traversing probability was lower and the traversing time was longer. Fig 6(b) shows the cell locations before and after traversing to the bottom trench layer. Cells got stuck in the pores when the trench depth was 3 or 6 μm. For larger trench depth of 15 or 30 μm, cells could traverse through the pores and migrate to the bottom trench layer. However, deeper trench of 30 μm lowered the traversing probability. Fig 6(c) shows the cell protrusion length distribution and most of the protrusion length was less than 30 μm. Therefore, less cells could reach the bottom surface of 30 μm deep trenches with their protrusions, resulting in lower probability to traverse through the porous membrane.

The findings above show that it was more difficult for NPC43 cells to traverse into trenches with shallower trench depth as indicated by the lower traversing probability or longer traversing time. Only a smaller number of NPC43 cells was able to undergo the large deformation to traverse through the 10 μm dia. pores into the 3 μm deep trenches. This is consistent with previous study that longer time was needed for metastatic cells to transit through narrower channels during the initial deformation [34]. In this study, only a few NPC43 cells could traverse to the bottom trench and migrate back to the top layer through another pore, while more cells could migrate back to the top layer through the same pore. When two NPC43 cells contacted each other and migrated around a pore, sometimes they would cluster together and traverse through the pore. However, cells that interacted with one another were not included in the analysis. In future study, it will be useful to compare cells that interact with other cells as well as different types of cells in the 3D platforms.

### Surface treatments on 3D platforms controlled cell traversing probability

**Fig 7:**
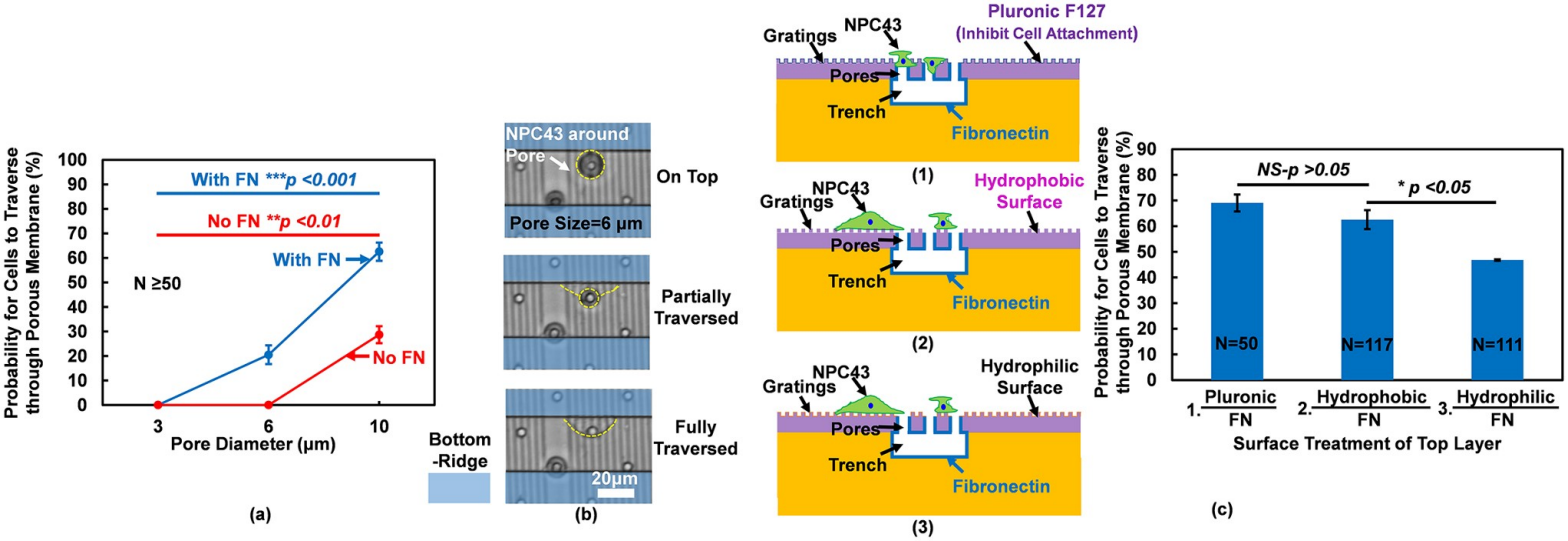
Effect of surface treatment on cell traversing probability. (a) Cells traversed through pores with dia. ranging from 3 to 10 μm with and without FN coating and top layer surface was hydrophobic. (b) Cell deformation when traversed through 6 μm dia. pore. (c) Surface treatments of top layer changed cell traversing probability. 2/2 μm trench/ridge and 1 μm deep gratings on top, 3, 6, 10 μm dia. and 14 μm deep pores in middle, and 30 μm wide and 15 μm deep trenches in bottom. 6 runs for 10 μm dia. pores without FN coating, 2 runs for 3/6 μm dia. pores without FN coating, and 3 runs for other conditions. **p <0.01 for effect of pore size on traversing probability without FN coating, ***p <0.001 for effect of pore size on traversing probability with FN coating, and *p <0.05 for traversing probability dependence on platforms with hydrophobic and hydrophilic coatings on top.

In order to increase cell traversing probability, surface treatments on three different layers of the 3-D platforms were performed. S1 Fig shows successful surface treatments by imaging FN on different layers. Coating of FN was on the pore sidewalls and bottom trenches, and not on the top surface. As shown in Fig 7(a), when pore size was 10 μm, the cell traversing probability almost doubled with FN coated on the pore sidewalls and bottom trenches while keeping top surface layer hydrophobic compared to platforms without surface treatment. 6 runs were carried out for 10 μm dia. pores without FN coating, 2 runs for 3/6 μm dia. pores without FN coating, and 3 runs for other conditions. With such coating, the smallest pore size cells could traverse decreased from 10 to 6 μm.

Fig 7(b) shows the cell morphology during the traversing process through 6 μm dia. pores with FN treatment. On the top layer before traversing, cell morphology was round and the migration mode was amoeboid. As the cell started to traverse through the pore, cell size decreased to squeeze into the pore, and the traversing process proceeded. After the cell traversed to the bottom trench layer, the cell became larger and elongated with protrusions from the cell body, and the cell switched to the mesenchymal migration mode along the trench sidewalls. Thus, the NPC43 cells went from amoeboid-to-mesenchymal migration mode when they traversed through the porous membrane with pore size that was smaller than cell size.

Different chemical coatings were applied on different layers of the 3D platforms as shown in Fig 7(c). 3 runs were carried out for all the conditions. Higher cell traversing probability occurred when Pluronic was coated on the top surface layer and FN was coated in the middle and bottom layers. As Pluronic inhibited cells from attaching on the top surface, they had higher probability to traverse through the pores to the trenches in the bottom layer. In addition, for comparison purpose, the top surface was made to be hydrophobic by not having any coating, and also to be hydrophilic by having an O_2_ plasma treatment as described earlier. There was still FN coating in the middle and bottom layers. Cell traversing probability decreased significantly when the top surface was hydrophilic. This was probably related to cells preferred to attach to the top surface instead of migrating into the bottom layer.

## Conclusions

In this paper, 3D platforms with different physical dimensions and topographies, as well as different chemical coatings were developed. The platform developed in this study was used to study cell migration through a porous membrane into a trench that mimicked a blood vessel. Higher cell traversing probability or shorter traversing time into pores with smaller size could be indications of higher metastatic potential. In addition, elements that influenced how cells squeezed through small pores included microstructure orientation with respect to trench sidewalls, pore shape and size, trench depth, and surface coating. NPC43 cell traversing behaviors through the pores were studied under different conditions. Cells had highest traversing probability when the 10 μm dia. pores were right next to the trench sidewalls below, as the sidewalls provided more surface area for cells to contact. Cell traversing probability increased when the top surface had guiding grating oriented perpendicular to the trenches below since the cells were guided to contact the trench sidewalls. In addition, cells changed from rounded to elongated shapes when they migrated circular pores with size smaller than cell size. Therefore, the cell migration mode might change from amoeboid to mesenchymal as the cell shape changed during cell traversing through pores.

Cells had protrusion length that was mostly less than 30 μm long. This resulted in 15 μm deep trenches to have higher cell traversing probability and shorter time needed for cells to traverse as cells could extend their protrusions to reach the bottom surface of the trenches. By coating the top surface with Pluronic and middle and bottom layers with FN, the smallest pore size that cells could traverse was reduced from 10 to 6 μm dia. Overall, coatings that made the top surface hydrophilic would keep the cells attached to the top surface, and lowered the cell traversing probability through the pores into the bottom trenches.

Compared to other studies, most of them used hydrogel or collagen as 3D matrix, which could not provide control of surface topography, pore size, shape or location, or trench dimension [18–24]. The 3D biomimetic platforms developed in this work provided precise control of the physical and chemical properties of the platforms. They allowed important controlling parameters for cell traversing through porous membrane to be identified without manipulation of medium flow. Real-time information related to cell invasion behaviors including traversing probability, migration mode, and traversing time could be obtained in the developed complex 3D platforms with precisely controlled microenvironment.

## Supporting information

S1 Fig FN coating on different layers of 3D platform. FN coatings on different layers of 3D platform from top view as shown in third image of Fig 7(c). (a) FN coated all around trenches in bottom layer. (b) FN coated around pore sidewalls. (c) No FN on top surface. 2/2 μm trench/ridge and 1 μm deep gratings on top, 10 μm dia. and 14 μm deep pores in middle, and 30 μm wide and 15 μm deep trenches in bottom. 3 runs.
(PPTX)

S1 Video Cell movement on platforms with straight and bent gratings. Platforms with (a) straight grating and pores and (b) bent grating and pores. The platforms had 2/2 μm trench/ridge and 1 μm deep straight/bent gratings on top, 10 μm dia. and 14 μm deep pores in middle.
(PPTX)
