# Supplementary material for: Traversing behavior of tumor cells in three-dimensional platforms with different topography
Source: PLoS One. 2020 Jun 10;15(6):e0234482. doi: 10.1371/journal.pone.0234482 (PMC7286507; doi:10.1371/journal.pone.0234482)
Supplement: S1 Fig — FN coatings on different layers of 3D platform from top view as shown in third image of Fig 7(c). (a) FN coated all around trenches in bottom layer. (b) FN coated around pore sidewalls. (c) No FN on top surface. 2/2 μm trench/ridge and 1 μm deep gratings on top, 10 μm dia. and 14 μm deep pores in middle, and 30 μm wide and 15 μm deep trenches in bottom. 3 runs. (PPTX) [file pone.0234482.s001.pptx]

## Slide 1
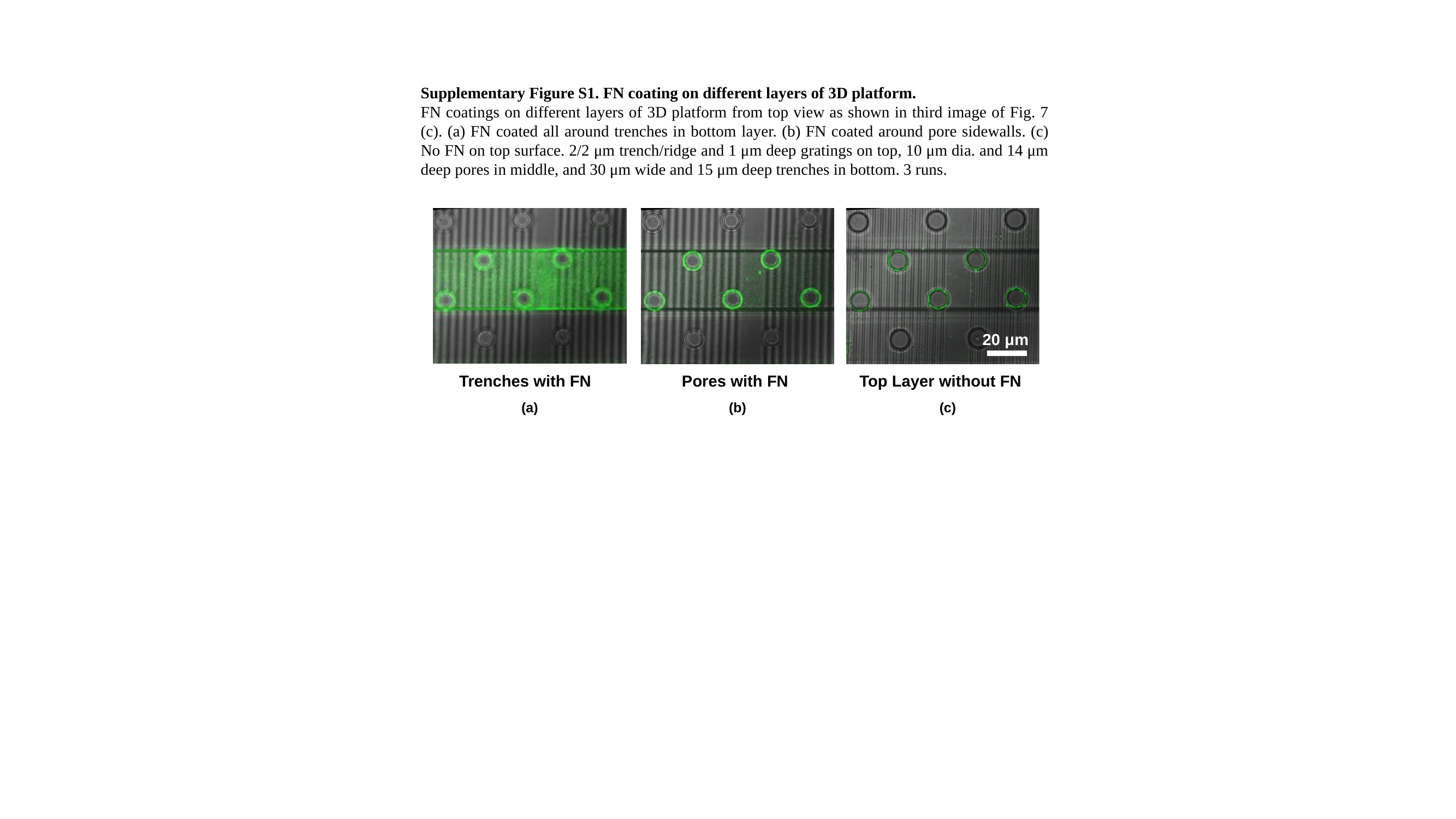

Supplementary Figure S1. FN coating on different layers of 3D platform.
FN coatings on different layers of 3D platform from top view as shown in third image of Fig. 7 (c). (a) FN coated all around trenches in bottom layer. (b) FN coated around pore sidewalls. (c) No FN on top surface. 2/2 μm trench/ridge and 1 μm deep gratings on top, 10 μm dia. and 14 μm deep pores in middle, and 30 μm wide and 15 μm deep trenches in bottom. 3 runs.
20 μm
Trenches with FN
Pores with FN
Top Layer without FN
(b)
(c)
(a)
