# Supplementary material for: Traversing behavior of tumor cells in three-dimensional platforms with different topography
Source: PLoS One. 2020 Jun 10;15(6):e0234482. doi: 10.1371/journal.pone.0234482 (PMC7286507; doi:10.1371/journal.pone.0234482)
Supplement: S1 Video — Platforms with (a) straight grating and pores and (b) bent grating and pores. The platforms had 2/2 μm trench/ridge and 1 μm deep straight/bent gratings on top, 10 μm dia. and 14 μm deep pores in middle. (PPTX) [file pone.0234482.s002.pptx]

## Slide 1
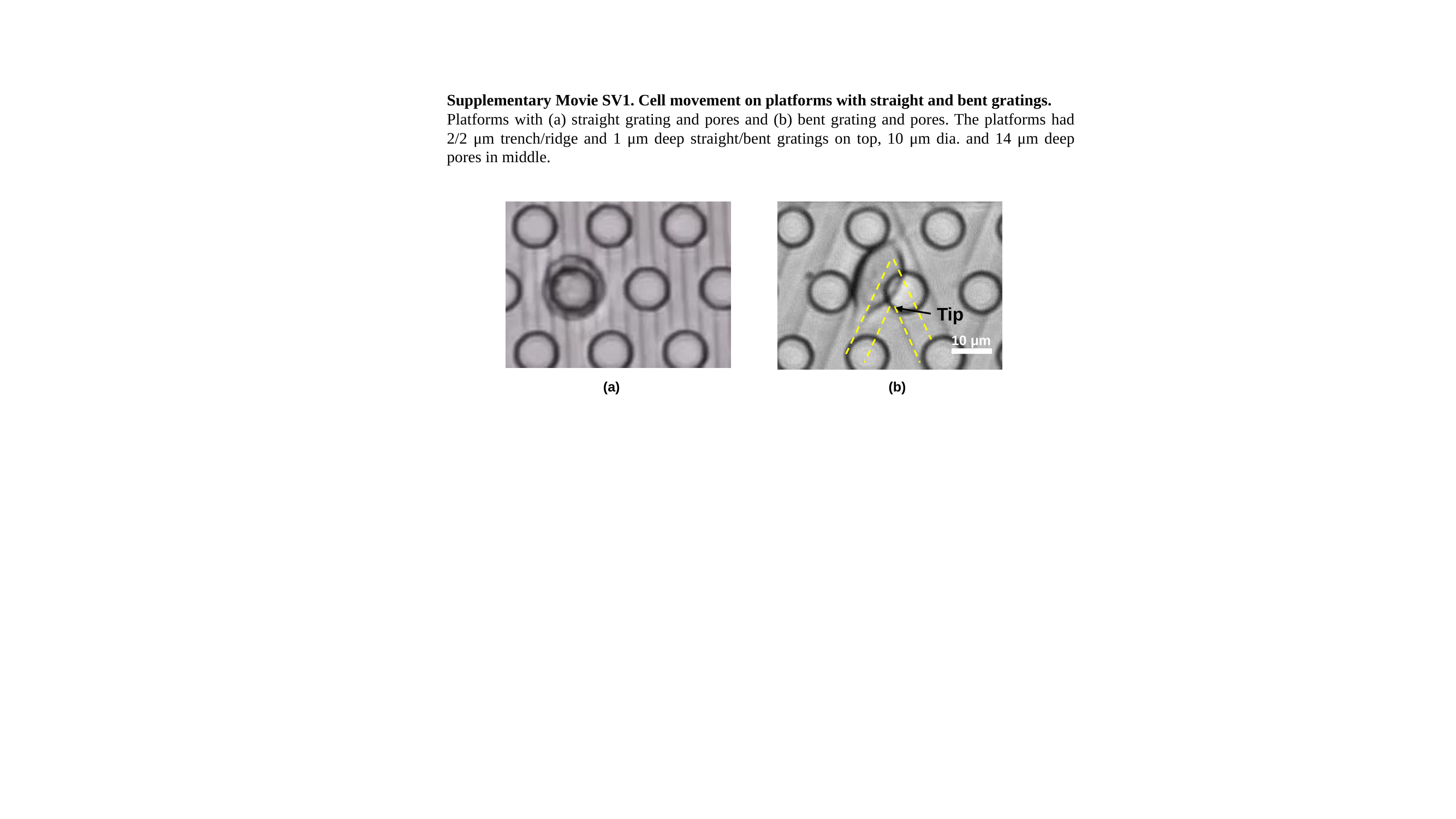

Supplementary Movie SV1. Cell movement on platforms with straight and bent gratings.
Platforms with (a) straight grating and pores and (b) bent grating and pores. The platforms had 2/2 μm trench/ridge and 1 μm deep straight/bent gratings on top, 10 μm dia. and 14 μm deep pores in middle.
Tip
10 μm
(a)
(b)
